# First isolation and identification of *Brucella microti* in sheep and goats: new insights and implications for veterinary medicine

**DOI:** 10.3389/fmicb.2025.1656803

**Published:** 2025-08-21

**Authors:** L. Freddi, V. Djokic, A. Dremeau, M. Ribeiro, M. Berthaud, F. Bennasar, C. Pailhous, A. Lanterne, A. Ferreira Vicente, C. Ponsart

**Affiliations:** ^1^Animal Health Laboratory, EU/WOAH and National Reference Laboratory for Brucellosis, Anses/Paris-Est University, Maisons-Alfort, France; ^2^ANSES, Ploufragan-Plouzané Niort Laboratory, Zoopôle, Ploufragan, France; ^3^Departmental Laboratory of Haute-Garonne (LD31 EVA), Launaguet, France; ^4^DDETSPP de l’Aveyron, Bouran, France; ^5^DDPP des Pyrénées-Atlantiques, Pau, France

**Keywords:** *Brucella microti*, small ruminants, brucellosis, diagnostics, surveillance strategy

## Abstract

Many species from the genus *Brucella* are causative agents of the bacterial zoonosis brucellosis. Until recently, it was generally believed that these bacteria exhibit strict host specificity; however, recent findings suggest otherwise. *Brucella microti* is an atypical *Brucella* species, no threat to humans, with a broad host spectrum, primarily found in wildlife and rodents, and is the only *Brucella* species isolated from soil, aquatic environments, and frogs, suggesting its environmental persistence and adaptability to diverse ecological niches. Despite its environmental resilience and wide host range, *B. microti* has not been detected in domestic animals. This study, for the first time, shows the ability of *B. microti* to infect domestic small ruminants. During the 2024 prophylaxis campaigns across three farms in two French departments, two sheep and one goat tested positive on classical serological tests for brucellosis. Following bacteriological isolation, HRM-PCR and classical biotyping methods classified the strains as *B. microti*, rather than the expected zoonotic *Brucella* spp. (*B. abortus*, *B. suis*, and *B. melitensis*). Hybrid whole-genome sequencing, whole genome single nucleotide polymorphism (wgSNP), and multiple Loci variable-number tandem repeat analysis (MLVA) revealed that the three isolates were genetically closer to the reference *B. microti* CCM4915 strains, isolated in Central Europe, than previously detected French strains from farmed frogs. The infection of small ruminants by *B. microti* is even more unusual, as no strain-specific antimicrobial resistance or virulence genes were identified. These findings underscore the need for new diagnostic tools that can identify *Brucellae* on the species level for proper management and monitoring, particularly in regions with epizootic risks. Further research is essential to clarify the role of *B. microti* in animal health and risks for public health.

## Introduction

Brucellosis is a bacterial zoonotic disease caused by gram-negative bacteria of the *Brucella* genus, usually transmitted from livestock to humans via the consumption of raw and unpasteurized animal food products. The disease remains a major global public health concern, with an estimated 1.6 to 2.1 million new human cases annually (Laine et al., 2023).

The genus *Brucella* includes numerous species (LPSN: https://lpsn.dsmz.de/genus/brucella). For clarity, in this study, the term *Brucella* only includes a unique monophyletic clade (Leclercq et al., 2020), which maintains both IS*711* and *bcsp31* genes (Bounaadja et al., 2009; Aljanazreh et al., 2023; Sanjuan-Jimenez et al., 2017), that have been used for molecular diagnostics. Previously, the *Brucellae* carrying both IS*711* and *bcsp31* comprised 14 species of facultative intracellular bacteria. Six of these species were grouped as classical and recognized as members of the core clade basis on their pathogenicity, host preferences, and adherence to phenotypic characteristics, including *B. abortus* (cattle), *B. melitensis* (goats or sheep), *B. suis* (swine), *B. ovis* (sheep and goats), *B. canis* (dogs), and *B. neotomae* (desert rats) (Occhialini et al., 2022; Olsen and Palmer, 2014; Suárez-Esquivel et al., 2020; Whatmore and Foster, 2021). This clade additionally includes the species *B. ceti* and *B. pinnipedialis*, which have been described more recently and isolated from marine mammals (Cloeckaert et al., 2001; Foster et al., 2007; Orsini et al., 2022). In the last two decades, advances in field research, pathogen detection, and molecular typing have made it possible to identify new species in addition to the strains not yet classified (Occhialini et al., 2022; Suárez-Esquivel et al., 2020). Two new species, *B. amazoniensis* and *B. nosferati*, isolated from Brazilian gold miners working in the Amazon rainforest (About et al., 2023) and Costa Rican bats, respectively (Hernández-Mora et al., 2023), can be considered part of the core clade *Brucella* based on phylogenetic analyses. However, we still do not know how these species are transmitted, what the animal reservoirs are, or their evolution in relation to other *Brucella* species. Additionally, four *Brucella* species, including *B. microti* (prevalent in common voles, red foxes, and wild boars), *B. inopinata* (in humans), *B. papionis* (in baboons), and *B. vulpis* (in red foxes), were identified in a wide range of hosts. These species can be distinguished from classical *Brucellae* by atypical phenotype, which includes altered metabolism, higher metabolic activity, faster growth, different composition of lipopolysaccharide, and presence of flagella (Occhialini et al., 2022). Due to these differences, new strains can be classified as a typical *Brucella* species, thereby contributing to the increased diversity within this genus (Occhialini et al., 2022). At the same time, applying a second tier of classification based on genetic homology shows that *B. microti* and *B. papionis* group more closely with the classical core clade species owing to their higher genomic similarity, whereas other atypical species remain genetically distant and thus appropriately classified as non-core clade *Brucella* (Occhialini et al., 2022).

*B. microti* was the first atypical *Brucella* species to demonstrate a broad host spectrum, with notable occurrences in the common vole (*Microtus arvalis*) in the Czech Republic (Scholz et al., 2008), mandibular lymph nodes of red foxes (*Vulpes vulpes*) from Austria (Scholz et al., 2009), and wild boars (*Sus scrofa*) from Hungary (Rónai et al., 2015). In experimental infections, *B. microti* exhibited high pathogenic potential, causing death in murine models, similar to classical *Brucella* spp. known to affect humans, thereby highlighting zoonotic risks (Jiménez et al., 2010). To date, no confirmed human infections have been reported. However, there was a suspected case in which, based on the clinical course, a clear epidemiological link (a bite from an infected rodent), isolation of the pathogen from a sick vole, and a specific serological response in the human patient, led to the conclusion that the disease was likely caused by *B. microti* (Hubálek et al., 2023).

Furthermore, *B. microti* was the only *Brucella* species to be isolated from soil (Scholz et al., 2008), indicating its environmental persistence, which diverges from the facultative intracellular evolution of classical core clade *Brucella* spp. Additionally, *B. microti* was also isolated from aquatic environments (Jaÿ et al., 2020), and the first strain was recovered from frogs (*Pelophylax ridibundus*) raised for human consumption (Jaÿ et al., 2018), further emphasizing its ability to persist in diverse ecological niches. The innate ability of *B. microti* to withstand acidic environments, ranging from moderate to extreme pH levels (Occhialini et al., 2022; Damiano et al., 2015; Freddi et al., 2017; de la Garza-García et al., 2021; Occhialini et al., 2012), along with its adaptation to anoxic conditions (Freddi et al., 2023), as well as accelerated metabolism, is associated with enhanced nutrient utilization and enzymatic activities (Al Dahouk et al., 2010; Al Dahouk et al., 2012). Furthermore, heterogeneity in LPS genes may explain its adaptability to environmental conditions. This environmental resilience, alongside its presence in a wide range of mammalian hosts, underscores its open lifestyle, making it distinct from more host-restricted *Brucella* species. However, to date, to the best of our knowledge, no identification of *B. microti* has been reported in cattle, small ruminants, or pigs, highlighting a gap in understanding its potential to colonize livestock and raising questions about its true zoonotic potential. With this study, the ability of *B. microti* to infect small ruminants is confirmed, shedding light on the potential risks and the need for proper management and identification of this bacterium in animals, especially from epizootic regions.

## Materials and methods

### Bacterial cultivation and strain isolation

All collected samples were analyzed at the Department laboratory of Haute-Garonne (LDA31EVA, Launaguet, France) using routine bacteriological procedures in the local veterinary diagnostic laboratories, in accordance with the U47-105 normative and French safety regulations in force. Thus, during slaughter of seropositive animals, three pairs of lymph nodes were collected aseptically, out of which 10 g were homogenized and diluted in 1/2 to 1/5 ratios in phosphate-buffered saline solution (0.9% NaCl PBS). The homogenate was then plated onto four *Brucella* selective Farrell media. Two plates were incubated at 37°C with 5% CO_2,_ and two plates without CO_2_ were incubated for up to 10 days. The isolates suspected to be *Brucella* were transferred to the French National Reference Laboratory for Animal Brucellosis (ANSES, Maisons-Alfort, France) to confirm the *Brucella* genus and determine the species, by safety regulations.

### Phenotypic identification and characterization

Isolates were characterized using standard procedures, following the World Organization for Animal Health (WOAH) guidelines, in a BSL-3 facility. The strains were biotype based on colonial morphology, Gram staining, CO_2_ requirement, H_2_S production, oxidase and urease activity, growth on dyes (basic fuchsin and thionin), lysis by phages (Tb, Wb, Iz, R/C), and agglutination with monospecific sera (anti-A, anti-M, and anti-R).

### Serological analyses

The Rose Bengal Test (RBT) and Complement Fixation Test (CFT) were performed on sera following the WOAH guidelines. Both diagnostic tests detect antibodies against smooth *Brucella* spp. CFT results were expressed as a titer (ICFTU/mL) with a positivity threshold of 20 ICFTU/mL.

### Molecular analyses

Genomic DNA was extracted using the commercial QIAGEN QIAamp DNA minikit (QIAGEN, Germany) following the manufacturer’s instructions. The real-time PCR (qPCR) was performed using the commercial qualitative *Brucella* spp. detection kit, ID Gene™ *Brucella* spp. triplex (Innovative Diagnostics, Montpellier, France), which targets the specific IS*711* insertion sequence, according to the manufacturer’s instructions. The PCR mix already contains primers and probes in the kit, and the following PCR program was used: denaturation at 95°C for 2 min, followed by 40 cycles of amplification at 95°C for 10 s and hybridization and elongation at 60°C for 30 s. Multiple Locus Variable-number Tandem Repeat Analysis (MLVA)-16 (Le Flèche et al., 2006) and High-Resolution Melting (HRM)-PCR (Girault et al., 2022) analyses were performed according to previously published protocols (Al Dahouk et al., 2010; Al Dahouk et al., 2012). The individual DNA samples of three isolates were typed with the MLVA-16 panel single-plex PCR, and the agarose gel method was used for amplicon identification. Clustering and congruence analyses were conducted with BioNumerics 7.6.3 (BioMérieux), using data as a character dataset via the categorical distance coefficient and MST (Minimum Spanning Tree) method. A total of 127 MLVA-16 profiles, including core (*n* = 81) and non-core clade (*n* = 46) *Brucella* available in the MLVA database^1^ or relative publications, were used in the analyses (Supplementary Table S1).

The short and long reads whole genome sequencing (WGS) of isolated strains was performed using Illumina DNA Prep kit (Illumina) and Rapid Barcoding Kit 24 V14 (Oxford Nanopore), respectively. The sequencing runs were performed on NextSeq 2000 equipment (Illumina) at the ANSES sequencing platform facility (ANSES, Ploufragan-Plouzané-Niort laboratory, France) and on MinION using Flow Cell R10.4.1 at the French National Reference Laboratory for Animal Brucellosis (Laboratory for Animal Health, ANSES, Maisons-Alfort, France). Raw reads were trimmed using Trimmomatic 0.36 for Illumina data and Nanofilt 1.10 (De Coster et al., 2018) for MinION data to remove low-quality bases. For Illumina data, trimming was performed with the following parameters: leading 3, trailing 3, sliding window 4:15, and minlen 50. For MinION data, trimming was performed using the -q 10 parameter to remove reads with a quality score below 10. A hybrid *de novo* assembly, combining Illumina and MinION raw reads, was performed using Unicycler 0.5.0 (Wick et al., 2017) to produce a more accurate and complete assembly. Finally, QUAST 5.2.0 was used to assess assembly robustness by gathering extensive assembly statistics. The three assemblies generated during this study have been deposited in the European Nucleotide Archive (ENA) at EMBL-EBI under accession number PRJEB89093.^2^

The whole genome single nucleotide polymorphism (wgSNP) analysis was performed using BioNumerics version 7.6.3 (BioMérieux) to trace back the source of infection. The genome of *B. melitensis* 16 M was used as a reference for comparative analyses across the entire *Brucella* genus, while the genome of *B. microti* CCM4915 served as the reference for comparisons within the only *B. microti* species. A total of 49 available *Brucella* genome sequences, representing all known *Brucella* species of the core clade (*n* = 38) and non-core clade strains (*n* = 11), were used in this study for comparative analysis (Supplementary Table S1). Chimeric genomes of chromosomes 1 and 2 were generated to compare complete and draft genomes (Huang et al., 2012). A minimum set of position filters was applied on the SNP matrix: (i) contiguous SNPs were removed (if found in a 10 bp-window), (ii) with non-informative SNPs, (iii) a required minimum of 15-fold coverage for each SNP, and (iv) ambiguous (i.e., non-ACGT bases) and unreliable bases (i.e., Ns) were discarded. The refined SNP matrix was used to generate a maximum likelihood tree based on the General Time Reversible model with 200 repetitions for bootstrap using MEGA version 11.0.13 (Tamura et al., 2021).

To target genes and/or regions potentially involved in the AMR, plasmid identification, and virulence (VG), all available *B. microti* genomes were screened using Abricate version 1.0.1^3^ as described in previous research (Girault et al., 2024). In summary, Abricate was run with entries from seven databases: for antimicrobial resistance genes AMRFinderPlus (NCBI) (Feldgarden et al., 2019), Comprehensive Antibiotic Resistance Database (CARD) (Jia et al., 2017), ResFinder (Zankari et al., 2012), and MEGARes 2.00 (Doster et al., 2020), for the virulence genes virulence factor database (VFDB) (Chen et al., 2016), and in-house database (BRUgenes), while for plasmid presence, PlasmidFinder (Carattoli et al., 2014) was used.

## Results

### Farms description

The first farm, located in the Aveyron department of France, operates a dual livestock system, combining both cattle and sheep husbandry. The beef herd consists of around forty Aubrac or Aubrac-cross dairy cows, with a grazing system for calves. The dairy sheep flock regroups 750 to 800 Lacaune ewes, depending on the year. The milk from these ewes is collected for the production of local fresh cheese.

The second farm, a dairy sheep farm, located in the Pyrénées-Atlantiques department, has a mixed livestock population, including six breeding rams, 250 ewes (over 18 months), 15 lambs (6–18 months), 80 bovines, and 50 horses during summer pastures. The epidemiological investigation highlighted the presence of domestic pets (three cats and dogs) at the farm, along with possible contact of breeding animals with wildlife species such as wild boar, roe deer, hares, rabbits, foxes, vultures, and bats. Stray animals were not observed on the farm. The type of feed provided included hay, regrowth forage, and complementary feed (corn, alfalfa).

The third farm, a dairy goat farm located in the same Pyrénées-Atlantiques department, which practices hand milking, is exclusively dedicated to dairy production and comprises 42 adult goats (over 12 months) and 14 younger animals (including approximately two bucks). The epidemiological investigation noted regular exposure to wildlife, with sightings of roe deer, foxes, martens, wild boar, and various birds within a fenced wooded area. In addition, about twenty feral goats were observed. These animals had been gathered and restrained by local breeders and were destined for export to Spain at the end of December 2023. Furthermore, the farm operates a communal management system on a shared parcel together with three other local breeders. The feeding regimen consisted primarily of natural forages, including brambles and woodland vegetation, supplemented by corn.

### Indirect diagnostics

All indirect diagnostic tests were carried out during the routine national prophylaxis campaign conducted by laboratories in two different French departments in 2024. As part of the farm’s brucellosis control measures, a sample of 50 ewes was collected from the first farm in May. Only one animal resulted serologically positive on RBT and showed an inconclusive result on CFT due to an anti-complementary reaction, without any clinical symptoms evocative of brucellosis. Although it is necessary to repeat the diagnosis after six to 8 weeks, the sanitary authority decided to proceed with the diagnostic slaughter (investigative culling) of seropositive sheep to expedite the process.

On March 18th, blood samples were collected from 57 sheep at the second farm. Serological testing identified one positive sheep in RBT and CFT (with an antibody titer of 853 ICFTU/mL). A follow-up test on April 30th confirmed the seropositive status of the same animal, with a decreased CFT titer of 213 ICFTU/mL. In addition, the animal tested positive again on June 20^th^, with a significantly increased CFT titer of 1,707 ICFTU/mL, which reduces the likelihood of a false-positive serological result. The *Brucella* seropositive sheep showed no clinical signs related to Brucellosis infection.

As part of the same prophylaxis campaign in the Pyrénées-Atlantiques department, blood samples were collected from 47 goats on the third farm on April 29^th^. Of the 47 sera tested, only one was found to be positive on RBT and CFT, with an antibody titer of 26.6 ICFTU/mL. A confirmatory test conducted on June 14th reaffirmed the positive result, although the CFT titer had decreased to 20 ICFTU/mL. As with the sheep, the goat showed no signs of brucellosis infection.

### Bacterial isolation and molecular diagnostics

Following the national brucellosis control program, bacteriological investigations were initiated for three animals following the seropositive results. These investigations took place on May 28th at the first farm and on July 27th at two other farms. Three pairs of lymph nodes (retropharyngeal, genital, and retromammary) from each animal were collected and sent to an accredited Department laboratory of Haute-Garonne (LDA31EVA, Launaguet, France) for *Brucella* culture diagnosis. Following cultivation, four *Brucella* spp. suspect colonies were isolated from three animals. From genital lymph nodes, one colony was isolated from a sheep from the first farm and a goat from the third farm. From the retromammary lymph nodes, one colony originated from a sheep from the second farm and the goat from the third farm. To confirm the *Brucella* genus identification and determine the species, all four suspected strains were transferred to the French National Reference Laboratory for brucellosis. After total DNA extraction, the qPCR analysis amplified the IS*711 Brucella* gene. The targeted gene was amplified in all four strains, resulting in a positive signal indicating that the strains belong to the genus *Brucella*. When these DNAs were subsequently tested for rapid identification and differentiation of the *Brucella* genus in HRM-PCR, the melting curve profiles matched with *Brucella microti*, instead of the expected classical smooth *Brucella* species like *B. abortus*, *B. suis*, and *B. melitensis*, which were the first expected diagnosis, regarding the host species.

### Genomic and bacteriological identification and characterization

The standard bacteriological phenotypic identification of the four isolates (code numbers 24–6,286-7554 for the first farm, 24–6,283-7553 for the second farm, and 24–6,281-7551 and 24–6,281-7552 for the third farm) confirmed the presence of the *Brucella* genus, with biotyping traits consistent with *B. microti* (Table 1). In particular, the newly identified strains showed agglutination only with anti-M, but not with anti-A and anti-R monospecific sera, consistent with the reference strain *B. microti* CCM 4915. In contrast, the 2017 French frog isolate (17–2,122-4144) agglutinated with only anti-A monospecific sera (Table 1).

**Table 1:** Classical phenotypic characterization of the four isolated strains (code numbers 24–6,286-7554 for 1st farm, 24–6,281-7551 and 24–6,281-7552 for 2nd farm, and 24–6,283-7553 for 3rd farm) from the three farms, compared to the reference *B. microti* CMC4915 strain and *B. microti*-like 17–2,122-4144 isolated from the marsh frog.

	*B. microti* CMC4915	*B. microti-like* 17–2,122-4144	24–6,286-7554 (genital LN sheep 1st farm)	24–6,283-7553 (retromammary LN sheep 2nd farm)	24–6,281-7551 (genital LN goat 3rd farm)	24–6,281-7552 (retromammary LN goat 3rd farm)
Morphology	S	S	S	S	S	S
CO_2_	−	−	−	−	−	−
H_2_S	−	−	−	−	−	−
Oxidase	+	+	+	+	+	+
Urease	+ slow	+ slow	+ slow	+ slow	+ slow	+ slow
A	−	+	−	−	−	−
M	+	−	+	+	+	+
R	−	−	−	−	−	−
Thionin	+	+	+	+	+	+
Fuchsin	+	+	+	+	+	+
Tb RTD	−	−	−	−	−	−
Tb 10^4^ RTD	+	+	+	+	+	+
Wb RTD	+	+	+	+	+	+
Iz RTD	+	+	+	+	+	+
R/C RTD	−	−	−	−	−	−

R/S, Colony morphology (Rough/Smooth), CO^2^ requirement, H_2_S production; agglutination with monospecific A, M, and R (rough) antisera; Dye (thionin and basic fuchsin) concentration 20 μg/mL in serum dextrose medium (1/50,000); + = growth or Lysis by phages; − = no growth or lysis.

**Figure 1:**
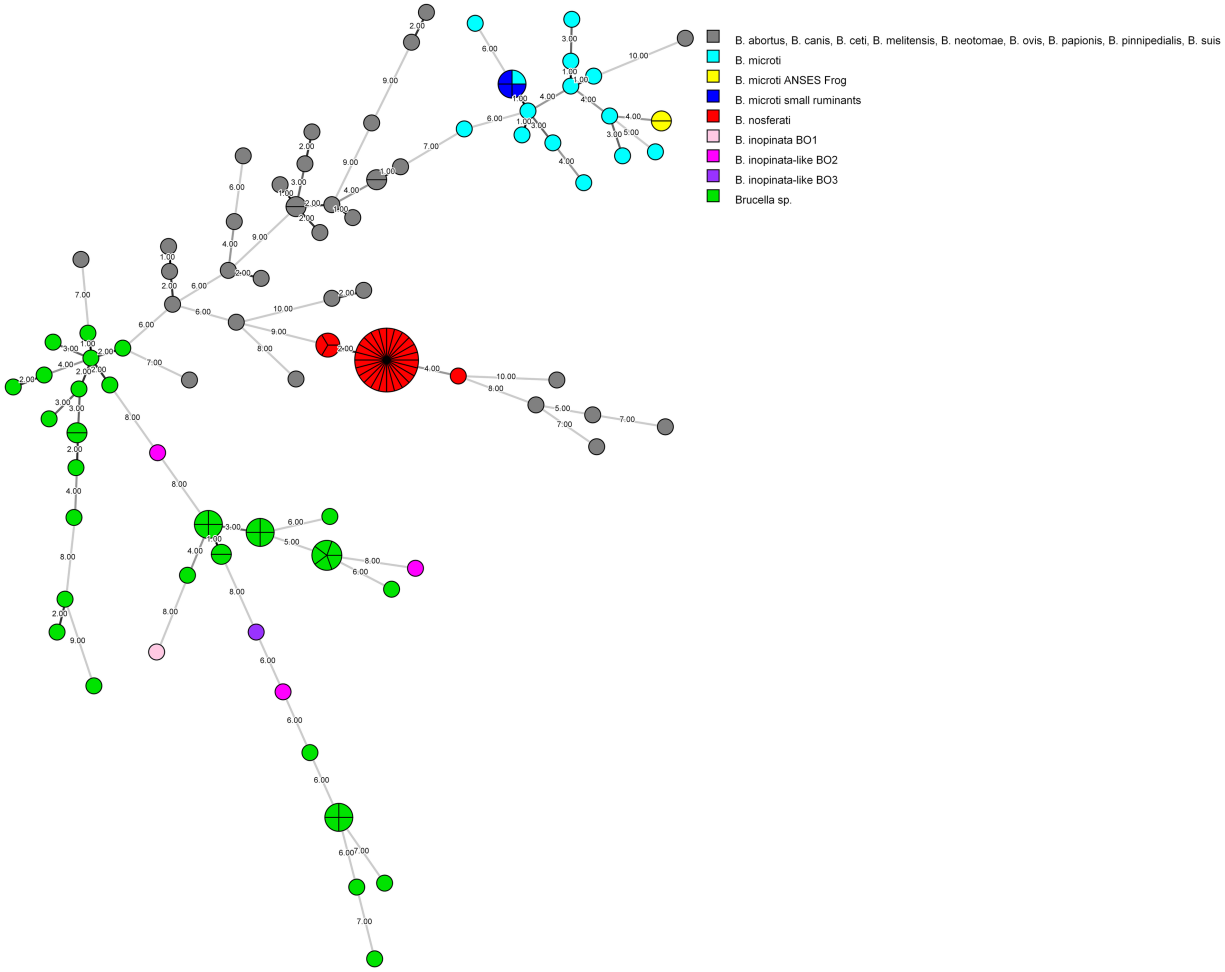
MLVA-16 minimum spanning tree describing relationships of strains investigated in this study, as well as all core (*n* = 81) and non-core clade (*n* = 46) *Brucella* species and strains. Clustering and partitioning were generated with BioNumerics, using data as a character dataset with a categorical distance coefficient and the minimum spanning tree method. Grey circles represent MLVA-16 genotypes of *B. abortus, B. canis, B. ceti, B. melitensis, B. neotomae, B. ovis, B. papionis, B. pinnipedialis, B. suis*, while others are colored with respect to available strains of *B. inopinata, B. inopinata-like, B. microti*, *B. nosferati,* and *Brucella* sp., including this study. The size of the circle indicates the number of strains corresponding to that genotype. The branch labels and numbers account correspond to the number of differing loci between nodes.

To genotype the strains and potentially determine the source of the infection, phylogenetic investigations were performed on one strain per animal and farm. The MLVA analysis confirmed the *B. microti* identity of three tested isolates from three farms (Figure 1). All three isolates (colored in blue) clustered together with the known *B. microti* strains (colored in turquoise) and, in particular, perfectly matched with *B. microti* CCM 4915, published by Audic et al. (2009) (Supplementary Figure 1).

**Figure 2:**
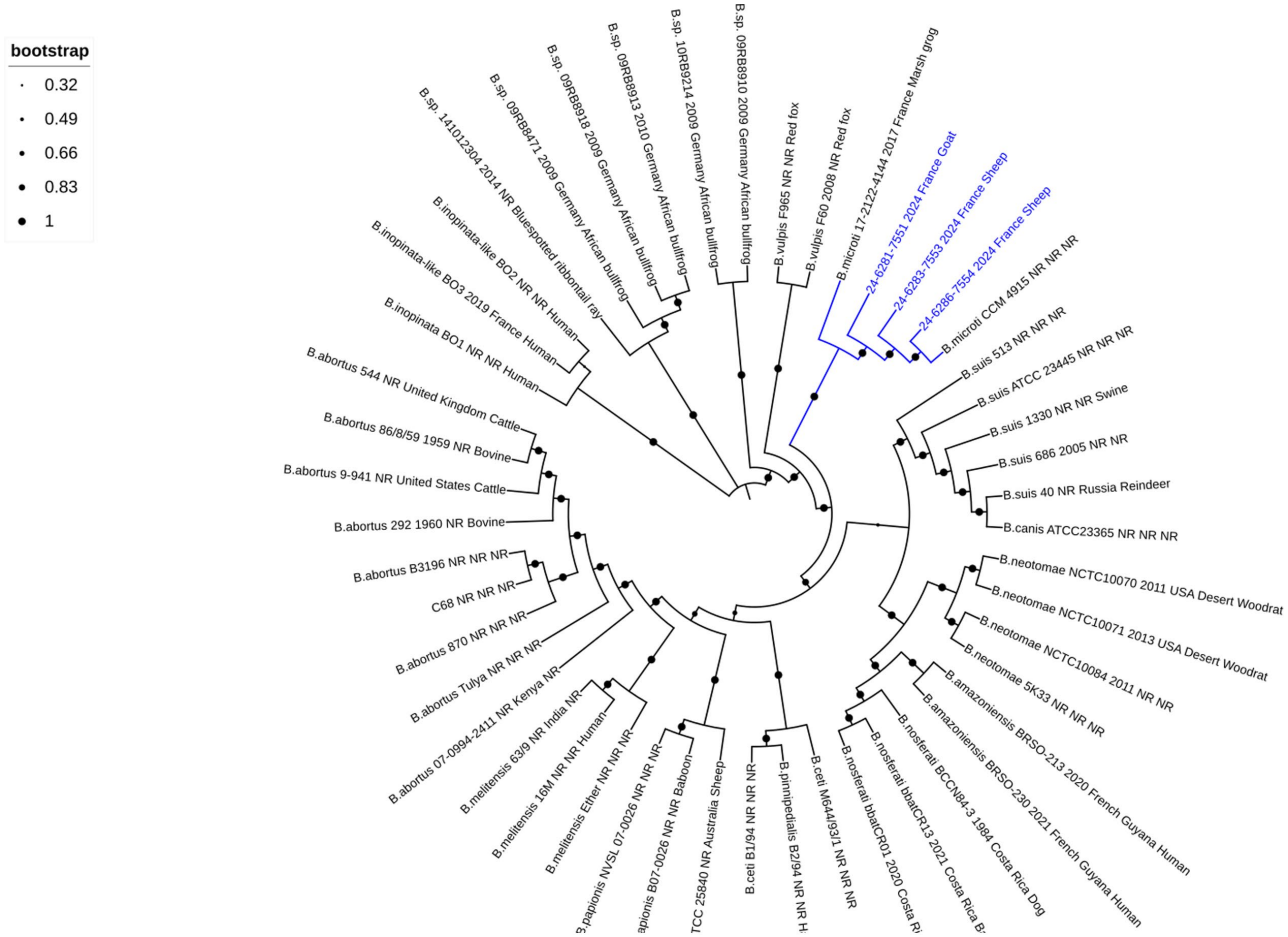
Phylogenetic comparative whole-genome SNP analysis of the small ruminant strains investigated in this study and all *Brucella* reference strains. The dendrogram was constructed using the maximum likelihood method with 200 bootstrap repetitions based on SNPs matrix (31′481 filtered SNPs). The phylogenetic tree was visualized with the EMBL online tool “Interactive Tree of Life” (iTOL v6) and annotated with four concatenated datasets separated based on underscores (*Brucella* species, strain name, year of isolation, isolation country, and host), colored in black and blue for all species and three isolates from small ruminants, respectively. The blue color was used to identify the *B. microti* branches. Not reported data are marked with “NR.” A log scale is used in the tree, allowing a better distinction between isolates.

To further characterize the strains, hybrid whole-genome sequencing using both long- and short-read methods was performed, aiming to obtain a high-precision assembly. In total, two contigs corresponding to the two chromosomes were identified for all three sequenced isolates. The total genome sizes were 3,338,075 bp, 3,338,083 bp, and 3,338,107 bp for isolates 24–6,281-7551, 24–6,283-7553, and 24–6,286-7554, respectively. The wgSNP analysis was conducted on available *Brucella* species sequences representing both core (*n* = 38) and non-core (*n* = 11) clades (Supplementary Table 1) to determine the genomic relationship of isolated strains. Phylogenetic comparative whole-genome SNP analysis showed that three strains clustered with other known *B. microti* strains (Figure 2). Notably, when using *B. melitensis* 16 M as the reference, out of 31,481 filtered SNPs, the two sheep isolates (24–6,286-7554 and 24–6,283-7553 from farms one and two, respectively) showed no differences compared to the reference *B. microti* CCM 4915 strain. In contrast, the goat isolate (24–6,281-7551 from the farm three) exhibited only one SNP difference, while the frog isolate (17–2,122-4144) showed 11 SNPs difference, compared to the CCM 4915 strain. When comparing only *B. microti* isolates aligned to the *B. microti* CCM 4915 reference genome, out of 146 filtered SNPs, the two sheep isolates (24–6,286-7554 and 24–6,283-7553 from farms one and two, respectively) displayed a difference of seven SNPs relative to the reference strain. The goat isolate (24–6,281-7551 from the farm three) exhibited eight SNPs, while the frog isolate (17–2,122-4144) showed differences in 142 SNPs compared to the CCM 4915 strain.

Finally, to better characterize the *B. microti* isolates from small ruminants and identify potential differences with other known *B. microti* species, an *in-silico* analysis was performed targeting the presence of plasmids, antimicrobial resistance (AMR), and virulence genes. No plasmids were detected in any of the *B. microti* strains when screening the assemblies against the PlasmidFinder database. Similarly, no antibiotic resistance genes were found when screening the NCBI and ResFinder databases. However, when searching through the CARD and MEGARes databases, six genes (*mprF, bep C*, *D*, *E*, *F*, and *G*) involved in AMR mechanisms were identified in all examined genomes, with a minimum identity percentage of 99.64%. Furthermore, all 53 *Brucella* spp. virulence genes were identified in the four *B. microti* genomes from this study as well as the previous frog isolate, with a minimal identity percentage of 97.08%, primarily associated with host immune evasion, intracellular survival, and the regulation and expression of the type IV secretion system in *Brucella* spp., based on the VFDB and BRUgenes databases.

## Discussion

Brucellosis is a highly contagious bacterial zoonosis, primarily transmitted to humans through the consumption of contaminated, unpasteurized dairy products, undercooked meat, or direct contact with infected livestock and their reproductive materials. The major species responsible for brucellosis in humans, *B. melitensis* and *B. abortus*, are typically associated with livestock, making comprehension of transmission paramount for control measures and public health protection. No *B. microti* human cases have been reported worldwide, even if a strong exposure to this bacterial species occurred in French farmed frogs destined for human consumption (Jaÿ et al., 2020; Jaÿ et al., 2018). In the case of *B. microti*, the transmission pathways are unknown. Given the growing recognition of *B. microti* as a pathogenic species and the presence of all 53 *Brucella* spp. known virulence genes, the absence of confirmed human infection remains surprising. Indeed, *B. microti* appears highly pathogenic in common voles and other rodents (Hubálek et al., 2007), as confirmed by experimental infections in murine models (Jiménez et al., 2010; Hubálek et al., 2007; Hanna et al., 2011; Ouahrani-Bettache et al., 2019). The absence of human cases might be partly explained by a lower pathogenicity in non-rodent species (Rudolf et al., 2024). In frogs, only a low percentage of animals showed clinical symptoms (Jaÿ et al., 2020; Jaÿ et al., 2018), as well as in foxes (Scholz et al., 2009) and wild boars (Rónai et al., 2015). According to these data, *B. microti* is likely to behave as an opportunistic soil bacterium that occasionally infects mammals (other than rodents) through the ingestion of contaminated products, with limited potential for sustained transmission in these hosts (Rudolf et al., 2024).

Our study reports for the first time that *B. microti* is capable of infecting livestock, particularly small ruminants, thereby expanding the known host range of this emerging pathogen. *B. microti* exhibits several physiological characteristics, in particular, expanded metabolic activity, compared to classical zoonotic *Brucella* spp. This enhanced metabolic flexibility suggests a greater ability to survive and proliferate in environmental reservoirs. This may also contribute to the capacity of *B. microti* to infect a broader range of hosts. Therefore, host hopping could represent a key mechanism driving the spread of these epizootic species (Occhialini et al., 2022). While *B. microti* has been primarily considered as a pathogenic bacterium for rodents and wildlife, the potential for transmission to livestock has not been thoroughly investigated (Rudolf et al., 2024). The detection of *B. microti* in livestock may represent a previously overlooked link in the potential transmission pathways, bridging the gap between environmental reservoirs, wildlife, and domestic animals. Cross-species spill-over transmission is strongly influenced by the frequency, duration, and nature of contacts. Opportunities for human intervention strongly impact this parameter (Lloyd-Smith et al., 2009). In Europe, due to the increased size of some wildlife populations in agricultural areas, such as ungulates or suidae, together with a progressive reduction of the usable agricultural area (Perpiña et al., n.d.), the probability of contact between domestic and wild species has increased during the past decades (Ledger et al., 2022). This may lead to potential emergence of wildlife pathogens, as previously observed in domestic dogs infected with *B. suis* biovar 2, strains presenting high genomic similarities with those circulating in hares and wild boars (Girault et al., 2023).

At the same time, the fact that only one animal per farm was infected may indicate that either these infections were accidental, suggesting the opportunistic nature of *B. microti*, or that the current surveillance system can identify early onsets of farm infection and therefore protect against greater spread and economic losses. It is important to note that no total culling of the herds was carried out as recommended for control of zoonotic *Brucella* (*B. abortus*, *B. melitensis,* and *B. suis*), but only the positive animal was removed, and at present, no other positive serological results have been observed. These data suggest that the *B. microti* infections observed in small ruminants are accidental, indicating the opportunistic nature of infection, similar to *Ochrobactrum* spp. (Ryan and Pembroke, 2020; Thoma et al., 2009), rather than indicative of widespread transmission within herds, like those of classical *Brucella* species (Corbel et al., 2006; Godfroid et al., 2013). This raises the need to evolve current regulations by considering all *Brucella* species, beyond just the controlled zoonotic ones, and defining the appropriate measures based on the bacterial species-host combination. Nevertheless, the performance characteristics of the CFT and RBT tests in ruminants infected with *B. microti* have not been established. Therefore, the absence of detectable antibodies or intermittent and short-term seropositivity in seronegative animals cannot be excluded. Experimental infection studies in ruminants with *B. microti* would be valuable to generate validation data for these serological assays, as well as to explore pathogenicity in these hosts, since no clinical symptoms have been observed in these three cases.

The detection of *B. microti* in small ruminants underscores a significant challenge in current brucellosis monitoring strategies. Traditional serological tests, commonly used to detect classical *Brucella* infections in livestock, rely on the detection of antibodies against the lipopolysaccharides (LPS), specifically the O-polysaccharide (OPS) component, which links to the outer core and extends into the extracellular environment (Erridge et al., 2002; Mancilla, 2015). These OPS components are found in all smooth *Brucella* spp., where the relative abundance and distribution of a homopolymeric linear chain of N-formyl-perosamine residues, linked via *α*1,2 and/or α-1,3 glycosidic bonds, are responsible for the structure and antigenicity (Bundle et al., 1989; Moriyón and López-Goñi, 1998; Zygmunt et al., 2015). Although there are slight structural variabilities in the OPS, they are not crucial in the indirect diagnosis of *Brucella* infections, since anti-OPS antibodies recognize stable terminal sugar residues. However, this homogeneity in the OPS structure between *B. microti* and the surveyed highly zoonotic *Brucella* (*B. abortus*, *B. melitensis,* and *B. suis*) poses a significant challenge for diagnostics, as they share similar antigenic profiles, leading to cross-reactivity in serological tests. As a result, control plans based solely on immuno-serology may fail to accurately identify *B. microti*-infected animals. Currently, in the EU, ruminants infected with highly zoonotic *Brucella* sp. have to be slaughtered (Regulation EU 2020/689; ELI: http://data.europa.eu/eli/reg_del/2020/689/oj). In some member states like France, the whole cattle and small ruminant farm has to be slaughtered, which leads to significant economic losses for farmers and low acceptability for citizens. Therefore, the misdiagnosed infection with *B. microti* may lead to unnecessary culling of healthy livestock. Moreover, spontaneous mutations in *B. microti* can lead to the conversion of smooth LPS to a truncated rough form as previously identified (Ouahrani-Bettache et al., 2019). The absence of OPS in these rough strains makes the use of classical smooth *Brucella* sp. antigens ineffective for diagnostics, as is the case with rough strains of *B. canis* and *B. ovis* (Djokic et al., 2023). As envisaged by Ouahrani-Bettache et al. (Moriyón et al., 2004) the rough *B. microti* strain could serve as an interesting candidate for brucellosis vaccination in addition to existing vaccines, since it does elicit an antibody response that is distinguishable from that induced during active infection when complete OPS is present.

France has been officially recognized as brucellosis-free since 2005, following the European regulation (EFSA, 2023). In both departments affected by *B. microti* infection in small ruminants, no brucellosis outbreaks in cattle, sheep, or goats have been identified since 2003. Only sporadic cases of *B. suis* biovar two infection have been reported in suidae (Wendling et al., 2020). However, no data are available concerning *B. microti* prevalence and distribution in France, nor have accidental isolations been reported to the national reference laboratory aside from the previously described frog cases (Jaÿ et al., 2020; Jaÿ et al., 2018). The detection of *B. microti* in sheep and goats from three geographically distinct French farms highlights the potential for localized outbreaks. Moreover, the fact that these outbreaks occurred in different regions within the same timeframe suggests that *B. microti* may be more widespread than initially thought. Building on the previous study (Jaÿ et al., 2020), which reported a significant presence of *B. microti*-like strains in both domestic frogs (*Pelophylax ridibundus*) and surrounding environments, including water and soil, this study suggests that infection of small ruminants may be linked to environmental reservoirs of this pathogen, even in geographically distant areas, as the affected farms were located in different regions of France. The widespread environmental presence of *B. microti* in amphibian habitats raises the possibility that similar reservoirs may exist in agricultural environments, potentially facilitating transmission of the pathogen to livestock. The broad-spectrum wgSNP analysis linked the isolated strains from small ruminants in the same subclade with all known *B. microti* strains (Figure 2). However, there is a greater genetic similarity of these strains with the reference *B. microti* CCM 4915 isolated in Central Europe (Audic et al., 2009), compared to the frog isolates found in France. Interestingly, compared to *the B. microti* CCM 4915 reference strain, the two *B. microti* isolates from sheep in two different French departments exhibited no difference and variations between seven SNPs when aligned with *B. melitensis* 16 M and *B. microti* CCM 4915, respectively. In contrast, one and eight SNPs were detected in the goat isolate. These minimal genetic variations among the livestock isolates suggest a high degree of genetic similarity within this population. However, when compared to the frog isolate 17–2,122-4144, a more pronounced divergence was observed, with at least 10 and 134 SNPs distinguishing it from new isolates and *B. microti* CCM 4915. This genetic difference is also evident in MLVA-16 analysis, where the frog isolate (colored in yellow) clusters separately from the small ruminant strains (colored in blue), which group with *B. microti* CCM4915 (colored in turquoise) (Figure 1 and Supplementary Figure 1). This indicates that the frog isolate represents a possible distinct lineage, potentially reflecting ecological adaptation or host-specific evolution. The considerable variation of SNPs between the frog and livestock isolates underscores the genetic diversity within *B. microti*. It highlights the need for further investigation into the ecological and host-associated factors contributing to this diversity.

*B. microti* was isolated from reproductive tissues, including genital and retromammary lymph nodes, which raises the possibility of bacterial excretion in milk. Although milk samples were not available for direct testing, the presence of the bacterium in these tissues suggests a potential downstream risk associated with the consumption of unpasteurized dairy products from the implicated farms. This underscores the need for strict food safety practices and enhanced public health surveillance in such settings. A further limitation of this study is the inability to conduct environmental sampling in the surrounding areas of the farms or in wildlife habitats to trace the potential source of infection. Given that *B. microti* has been isolated from wild animals and soil, the possibility of environmental reservoirs contributing to the transmission cycle is high. Interestingly, the first two farms have 40 and 80 cattle, respectively, and no positive serological results were found during the prophylaxis campaigns before and after slaughter of infected ruminants. Additional annual monitoring of these herds has not shown any positive serological results. This suggests that, potentially, *B. microti*, at least for now, is more adapted to small ruminants. Identification of *B. microti* in domestic animals emphasizes the importance of monitoring *Brucella* infections across diverse populations, applying “One Health” approaches at the wildlife-farm-human interface (Lloyd-Smith et al., 2009). Future studies incorporating environmental sampling and broader surveillance of wildlife populations are essential to elucidate the transmission dynamics of this emerging pathogen and to devise effective control measures. Furthermore, the ability of *B. microti* to persist in wildlife and spill over into domestic animals suggests that targeted strategies are needed to prevent its transmission between these populations. Biosecurity is essential in livestock farming to prevent the spread of diseases, ensure animal welfare, and maintain farm sustainability (Bellini, 2018). In practice, to prevent various infectious diseases, it is recommended to maintain the surroundings of the farm double-fenced, which greatly limits the contact of wild animals with livestock. At the same time, it is recommended to avoid watering in ponds or rivers accessible to wildlife or downstream from other farms. Take precautions when distributing feed in pastures and restrict access to manure in the fields by using a tarpaulin or electric fencing. Domestic carnivores, suidae, and poultry can be sources of many infectious agents for cattle. In extensive and small-scale ruminant farms, biosecurity implementation may be impaired by inadequate premises infrastructure and uncontrolled contacts among different species (Alavedra et al., 2025). Compliance, in these cases, was influenced by farmers’ age, education level, herd size, and production.

In particular, dairy farms showed better biosecurity practices, probably because of improved management and infrastructure. This study highlights the challenges of implementing biosecurity measures on small-scale, extensive farms and shows the ineffectiveness of standardized plans. Biosecurity management in cattle farms consists of so-called protective measures to avoid the introduction of pathogens into the farm and limit the spread and the clinical expression of conditions already present in the farm. It includes a forward flow, management of introductions (animals, feed, workers, and instruments). Their presence should be prohibited in breeding and professional areas where feed is stored. This is true of dogs about neosporosis and poultry about botulism and salmonellosis. Finally, the installation of nets must prevent bird access to open-air feed storage to reduce the risk of contamination of milk by pathogens [*Salmonella*, HP STEC (highly pathogenic Shiga toxin-producing *Escherichia coli*), etc.], particularly in the case of raw milk production.

In conclusion, current results in conjunction with previous findings showed that *B. microti* is a potential pathogen at the interface of livestock, wildlife, and the environment. This highlights the need to improve screening tools for ruminants and to establish surveillance in wildlife and environmental reservoirs to better detect atypical *Brucella* species.

## Data Availability

The datasets presented in this study can be found in online repositories. The names of the repository/repositories and accession number(s) can be found in the article/Supplementary material.

## Footnotes

^1^ https://microbesgenotyping.i2bc.paris-saclay.fr/

^2^ https://www.ebi.ac.uk/ena/browser/view/PRJEB89093

^3^ https://github.com/tseemann/abricate
