# Supplementary material for: First isolation and identification of Brucella microti in sheep and goats: new insights and implications for veterinary medicine
Source: Front Microbiol. 2025 Aug 21;16:1656803. doi: 10.3389/fmicb.2025.1656803 (PMC12408521; doi:10.3389/fmicb.2025.1656803)
Supplement: Supplementary file 2 [file Data_Sheet_1.PDF]

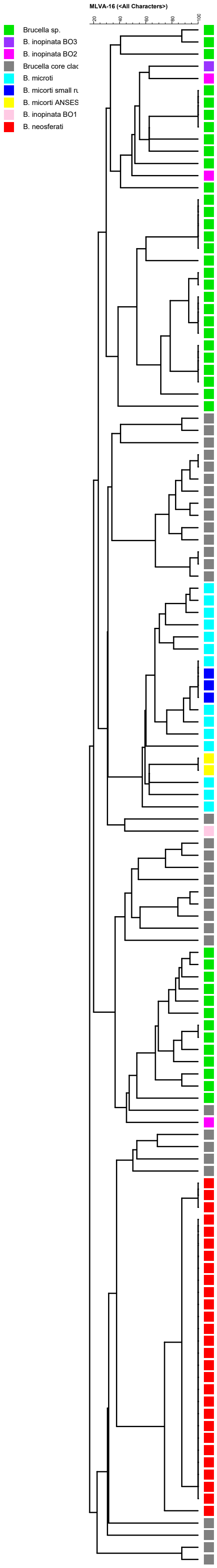

Brucella sp.	1315	Brucella spp.	Ceratophrys ornata		GCA_024970695 (https://www.ncbi.nlm.nih.gov/assembly/GCA_0249706
Brucella sp.	2594	Brucella spp.	Ceratophrys ornata		GCA_024970895 (https://www.ncbi.nlm.nih.gov/assembly/GCA_0249708
Brucella sp.	458	Brucella spp.	Homo sapiens		GCA_017797885 (https://www.ncbi.nlm.nih.gov/assembly/GCA_0177978
B. inopinata BO3	BO3	Brucella spp. BO3	Human		GCA_014084005 (https://www.ncbi.nlm.nih.gov/assembly/GCA_0140840
B. inopinata BO2	M6687	Brucella spp.	Homo sapiens		GCA_015832115 (https://www.ncbi.nlm.nih.gov/assembly/GCA_0158321
Brucella sp.	09RB8913	Brucella sp.	African bullfrog	Tanzania (Germany)	Al Dahouk2017(https://www.ncbi.nlm.nih.gov/pubmed/28300153)
Brucella sp.	09RB8914	Brucella sp.	African bullfrog	Tanzania (Germany)	Al Dahouk2017(https://www.ncbi.nlm.nih.gov/pubmed/28300153)
Brucella sp.	09RB8915	Brucella sp.	African bullfrog	Tanzania (Germany)	Al Dahouk2017(https://www.ncbi.nlm.nih.gov/pubmed/28300153)
Brucella sp.	09RB8918	Brucella sp.	African bullfrog	Tanzania (Germany)	Al Dahouk2017(https://www.ncbi.nlm.nih.gov/pubmed/28300153)
Brucella sp.	141012304	Brucella spp. (ribbontail ray)	Taeniura lymma (zoo)		GCA_900095155 (https://www.ncbi.nlm.nih.gov/assembly/GCA_9000951
Brucella sp.	2280	Brucella spp. (2280)	Human		GCA_009601725 (https://www.ncbi.nlm.nih.gov/assembly/GCA_0096017
Brucella sp.	2716	Brucella spp.	Megophrys nasuta		GCA_024970345 (https://www.ncbi.nlm.nih.gov/assembly/GCA_0249703
B. inopinata BO2	6810	Brucella sp.BO2	Homo sapiens		GCA_014495905 (https://www.ncbi.nlm.nih.gov/assembly/GCA_0144959
Brucella sp.	10RB9215	Brucella sp.	African bullfrog	Tanzania (Germany)	Al Dahouk2017(https://www.ncbi.nlm.nih.gov/pubmed/28300153)
Brucella sp.	09RB8908	Brucella sp.	African bullfrog	Germany	Scholz et al., 2016; Al Dahouk et al., 2017
Brucella sp.	09RB8909	Brucella sp.	African bullfrog	Germany	Scholz et al., 2016; Al Dahouk et al., 2017
Brucella sp.	09RB8910	Brucella sp.	African bullfrog	Germany	Scholz et al., 2016; Al Dahouk et al., 2017
Brucella sp.	10RB9211	Brucella sp.	African bullfrog	Germany	Scholz et al., 2016; Al Dahouk et al., 2017
Brucella sp.	10RB9212	Brucella sp.	African bullfrog	Germany	Scholz et al., 2016; Al Dahouk et al., 2017
Brucella sp.	10RB9214	Brucella sp.	African bullfrog	Tanzania (Germany)	Al Dahouk2017(https://www.ncbi.nlm.nih.gov/pubmed/28300153)
Brucella sp.	10RB9216	Brucella sp.	African bullfrog	Germany	Scholz et al., 2016; Al Dahouk et al., 2017
Brucella sp.	10RB9216	Brucella sp.	African bullfrog	Tanzania (Germany)	Al Dahouk2017(https://www.ncbi.nlm.nih.gov/pubmed/28300153)
Brucella sp.	10RB9205	Brucella sp.	African bullfrog	Germany	Scholz et al., 2016; Al Dahouk et al., 2017
Brucella sp.	10RB9207	Brucella sp.	African bullfrog	Germany	Scholz et al., 2016; Al Dahouk et al., 2017
Brucella sp.	10RB9208	Brucella sp.	African bullfrog	Germany	Scholz et al., 2016; Al Dahouk et al., 2017
Brucella sp.	10RB9209	Brucella sp.	African bullfrog	Germany	Scholz et al., 2016; Al Dahouk et al., 2017
Brucella sp.	09RB8471	Brucella sp.	African bullfrog	Germany	Scholz et al., 2016; Al Dahouk et al., 2017
Brucella sp.	10RB9206	Brucella sp.	African bullfrog	Germany	Scholz et al., 2016; Al Dahouk et al., 2017
Brucella sp.	10RB9217	Brucella sp.	African bullfrog	Germany	Scholz et al., 2016; Al Dahouk et al., 2017
Brucella sp.	10RB9217	Brucella sp.	African bullfrog	Tanzania (Germany)	Al Dahouk2017(https://www.ncbi.nlm.nih.gov/pubmed/28300153)
Brucella sp.	10RB9210	Brucella sp.	African bullfrog	Germany	Scholz et al., 2016; Al Dahouk et al., 2017
Brucella sp.	10RB9213	Brucella sp.	African bullfrog	Germany	Scholz et al., 2016; Al Dahouk et al., 2017
Brucella core clade	F8/08-60(T)	B.papionis	Baboon	Tanzania	Whatmore2014 (http://www.ncbi.nlm.nih.gov/pubmed/25242540)
Brucella core clade	F8/08-61	B.papionis	Baboon	USA:Texas	Whatmore2014 (http://www.ncbi.nlm.nih.gov/pubmed/25242540)
Brucella core clade	REF 94-74	B. ceti	Ref. (Harbour porpoise)	Scotland	Le Flèche et al., 2006
Brucella core clade	bneohCR01	B.neotomae	Human	Costa Rica	SuÃ¡rez-Esquivel2017a (https://www.ncbi.nlm.nih.gov/pubmed/28518028
Brucella core clade	bneohCR02	B.neotomae	Human	Costa Rica	SuÃ¡rez-Esquivel2017a (https://www.ncbi.nlm.nih.gov/pubmed/28518028
Brucella core clade	BCCN R16#	B.neotomae	Rodent	USA	Ledwaba2019 (https://www.ncbi.nlm.nih.gov/pubmed/31107864)
Brucella core clade	REF 5K33	B. neotomae	Ref. (Desert rat)	USA	Le Flèche et al., 2006
Brucella core clade	BCCN#R34	B.neotomae	Rodent	USA:Utah desert	Vergnaud2018 (https://www.ncbi.nlm.nih.gov/pubmed/30050522)
Brucella core clade	BCCN#R40	B.neotomae	Rodent	USA:Utah desert	Vergnaud2018 (https://www.ncbi.nlm.nih.gov/pubmed/30050522)
Brucella core clade	BCCN#R35	B.neotomae	Rodent	USA:Utah desert	Vergnaud2018 (https://www.ncbi.nlm.nih.gov/pubmed/30050522)
Brucella core clade	BCCN#R39	B.neotomae	Rodent	USA:Utah desert	Vergnaud2018 (https://www.ncbi.nlm.nih.gov/pubmed/30050522)
Brucella core clade	BCCN#R36	B.neotomae	Rodent	USA:Utah desert	Vergnaud2018 (https://www.ncbi.nlm.nih.gov/pubmed/30050522)
Brucella core clade	BCCN#R38	B.neotomae	Rodent	USA:Utah desert	Vergnaud2018 (https://www.ncbi.nlm.nih.gov/pubmed/30050522)
Brucella core clade	BCCN#R37	B.neotomae	Rodent	USA:Utah desert	Vergnaud2018 (https://www.ncbi.nlm.nih.gov/pubmed/30050522)
B. microti	(IMB) 10-17	B. microti	Soil	Austria	Scholz et al., 2008b
B. microti	(IMB) 10-20	B. microti	Soil	Austria	Scholz et al., 2008b
B. microti	BMS 10	B. microti	Soil	Czech Republic	Al Dahouk et al., 2012
B. microti	F303M	B. microti	Red fox	Austria	Al Dahouk et al., 2012
B. microti	CCM4916 or (IMB) 124	B. microti	Common vole	Czech Republic	Scholz et al., 2008a
B. microti	(IMB) 257	B. microti	Red fox	Austria	Scholz et al., 2009
B. microti	CCM 4915	B. microti	Ref. (Common vole)	Czech Republic	Audic et al., 2009
B. micorti small ruminants	24-6286-7554	B. microti	sheep	France	This study
B. micorti small ruminants	24-6283-7553	B. microti	sheep	France	This study
B. micorti small ruminants	24-6281-7551	B. microti	goat	France	This study
B. microti	CCM4915 or (IMB) 122	B. microti	Ref. (Common vole)	Czech Republic	Scholz et al., 2008a
B. microti	CCM4915	B. microti	Ref. (Common vole)		Jaÿ at al., 218, https://doi.org/10.3389/fvets.2018.00283
B. microti	FK 21908	B. microti	Red fox	Austria	Al Dahouk et al., 2012
B. microti	FH 2208	B. microti	Red fox	Austria	Al Dahouk et al., 2012
B. micorti ANSES Frog	17-2122-1	B. microti	Marsh frog	France	Jaÿ at al., 218, https://doi.org/10.3389/fvets.2018.00283
B. micorti ANSES Frog	17-2122-2	B. microti	Marsh frog	France	Jaÿ at al., 218, https://doi.org/10.3389/fvets.2018.00283
B. microti	FW 70608	B. microti	Red fox	Austria	Al Dahouk et al., 2012
B. microti	(IMB) 284	B. microti	Red fox	Austria	Scholz et al., 2009
B. microti	HUN-Bmi-01	B. microti	Wild boar	Hungary	Ronai et al., 2015
Brucella core clade	REF B2-94	B. pinnipedialis	Ref. (Harbour seal)	Scotland	Le Flèche et al., 2006
B. inopinata BO1	REF BO1	B. inopinata	Ref. (Human)	USA	Scholz et al., 2010; Al Dahouk et al., 2012
Brucella core clade	REF 86-8-59	B. abortus bv 2	Ref. (Cattle)	England	Le Flèche et al., 2006
Brucella core clade	REF 292	B. abortus bv 4	Ref. (Cattle)	England	Le Flèche et al., 2006
Brucella core clade	REF 544	B. abortus bv 1	Ref. (Cattle)	England	Le Flèche et al., 2006
Brucella core clade	REF Tulya	B. abortus bv 3	Ref. (Human)	Uganda	Le Flèche et al., 2006
Brucella core clade	REF B3196	B. abortus bv 5	Ref. (Cattle)	England	Le Flèche et al., 2006
Brucella core clade	REF C68	B. abortus bv 9	Ref. (Cattle)	England	Le Flèche et al., 2006
Brucella core clade	REF 870	B. abortus bv 6	Ref. (Cattle)	Africa	Le Flèche et al., 2006
Brucella core clade	REF Ether	B. melitensis bv 3	Ref. (Goat)	Italia	Le Flèche et al., 2006
Brucella core clade	REF 63-9	B. melitensis bv 2	Ref. (Goat)	Turkey	Le Flèche et al., 2006
Brucella sp.	BCCN#10-4	Brucella spp.(Australian rodents)	Rodent	Australia	Vergnaud2018 (https://www.ncbi.nlm.nih.gov/pubmed/30050522)
Brucella sp.	BCCN#10-6	Brucella spp.(Australian rodents)	Rodent	Australia	Vergnaud2018 (https://www.ncbi.nlm.nih.gov/pubmed/30050522)
Brucella sp.	BCCN#10-2	Brucella spp.(Australian rodents)	Rodent	Australia	Vergnaud2018 (https://www.ncbi.nlm.nih.gov/pubmed/30050522)
Brucella sp.	BCCN#10-5	Brucella spp.(Australian rodents)	Rodent	Australia	Vergnaud2018 (https://www.ncbi.nlm.nih.gov/pubmed/30050522)
Brucella sp.	BCCN#10-8	Brucella spp.(Australian rodents)	Rodent	Australia	Vergnaud2018 (https://www.ncbi.nlm.nih.gov/pubmed/30050522)
Brucella sp.	BCCN#83-210	Brucella spp.(Australian rodents)	Rodent	Australia	Vergnaud2018 (https://www.ncbi.nlm.nih.gov/pubmed/30050522)
Brucella sp.	BCCN#10-10	Brucella spp.(Australian rodents)	Rodent	Australia	Vergnaud2018 (https://www.ncbi.nlm.nih.gov/pubmed/30050522)
Brucella sp.	BCCN#83-211	Brucella spp.(Australian rodents)	Rodent	Australia	Vergnaud2018 (https://www.ncbi.nlm.nih.gov/pubmed/30050522)
Brucella sp.	BCCN#10-11	Brucella spp.(Australian rodents)	Rodent	Australia	Vergnaud2018 (https://www.ncbi.nlm.nih.gov/pubmed/30050522)
Brucella sp.	BCCN#10-9	Brucella spp.(Australian rodents)	Rodent	Australia	Vergnaud2018 (https://www.ncbi.nlm.nih.gov/pubmed/30050522)
Brucella sp.	BCCN#10-1	Brucella spp.(Australian rodents)	Rodent	Australia	Vergnaud2018 (https://www.ncbi.nlm.nih.gov/pubmed/30050522)
Brucella sp.	BCCN#10-3	Brucella spp.(Australian rodents)	Rodent	Australia	Vergnaud2018 (https://www.ncbi.nlm.nih.gov/pubmed/30050522)
Brucella sp.	VLA-83/13	Brucella spp.(Australian rodents)	Rodent	Australia	SRR011179 (http://www.ncbi.nlm.nih.gov/nuccore/SRR011179)
Brucella core clade	REF 513	B. suis bv 5	Ref. (Wild rodent)	Former USSR	Le Flèche et al., 2006
B. inopinata BO2	BCCN#10-12	Brucella sp.BO2	Human	USA	Vergnaud2018 (https://www.ncbi.nlm.nih.gov/pubmed/30050522)
Brucella core clade	REF 40	B. suis bv 4	Ref. (Reindeer)	Former USSR	Le Flèche et al., 2006
Brucella core clade	REF RM 6-66	B. canis	Ref. (Dog)	United States	Le Flèche et al., 2006
Brucella core clade	REF 686	B. suis bv 3	Ref. (Swine)	USA	Le Flèche et al., 2006
Brucella core clade	REF 1330	B. suis bv 1	Ref. (Swine)	USA	Le Flèche et al., 2006
B. neosferati	bbatCR09	B. nosferati	Desmodus rotundus	Costa Rica	Hernández-Mora G et al., mSphere 8:e00061-23.https://doi.org/10.1128/r
B. neosferati	bbatCR10	B. nosferati	Desmodus rotundus	Costa Rica	Hernández-Mora G et al., mSphere 8:e00061-23.https://doi.org/10.1128/r
B. neosferati	bbatCR11	B. nosferati	Desmodus rotundus	Costa Rica	Hernández-Mora G et al., mSphere 8:e00061-23.https://doi.org/10.1128/r
B. neosferati	bbatCR01	B. nosferati	Desmodus rotundus	Costa Rica	Hernández-Mora G et al., mSphere 8:e00061-23.https://doi.org/10.1128/r
B. neosferati	bbatCR02	B. nosferati	Desmodus rotundus	Costa Rica	Hernández-Mora G et al., mSphere 8:e00061-23.https://doi.org/10.1128/r
B. neosferati	bbatCR03	B. nosferati	Desmodus rotundus	Costa Rica	Hernández-Mora G et al., mSphere 8:e00061-23.https://doi.org/10.1128/r
B. neosferati	bbatCR04	B. nosferati	Desmodus rotundus	Costa Rica	Hernández-Mora G et al., mSphere 8:e00061-23.https://doi.org/10.1128/r
B. neosferati	bbatCR05	B. nosferati	Desmodus rotundus	Costa Rica	Hernández-Mora G et al., mSphere 8:e00061-23.https://doi.org/10.1128/r
B. neosferati	bbatCR06	B. nosferati	Desmodus rotundus	Costa Rica	Hernández-Mora G et al., mSphere 8:e00061-23.https://doi.org/10.1128/r
B. neosferati	bbatCR07	B. nosferati	Desmodus rotundus	Costa Rica	Hernández-Mora G et al., mSphere 8:e00061-23.https://doi.org/10.1128/r
B. neosferati	bbatCR08	B. nosferati	Desmodus rotundus	Costa Rica	Hernández-Mora G et al., mSphere 8:e00061-23.https://doi.org/10.1128/r
B. neosferati	bbatCR24	B. nosferati	Desmodus rotundus	Costa Rica	Hernández-Mora G et al., mSphere 8:e00061-23.https://doi.org/10.1128/r
B. neosferati	bbatCR23	B. nosferati	Desmodus rotundus	Costa Rica	Hernández-Mora G et al., mSphere 8:e00061-23.https://doi.org/10.1128/r
B. neosferati	bbatCR19	B. nosferati	Desmodus rotundus	Costa Rica	Hernández-Mora G et al., mSphere 8:e00061-23.https://doi.org/10.1128/r
B. neosferati	bbatCR16	B. nosferati	Desmodus rotundus	Costa Rica	Hernández-Mora G et al., mSphere 8:e00061-23.https://doi.org/10.1128/r
B. neosferati	bbatCR13	B. nosferati	Desmodus rotundus	Costa Rica	Hernández-Mora G et al., mSphere 8:e00061-23.https://doi.org/10.1128/r
B. neosferati	bbatCR29	B. nosferati	Desmodus rotundus	Costa Rica	Hernández-Mora G et al., mSphere 8:e00061-23.https://doi.org/10.1128/r
B. neosferati	bbatCR28	B. nosferati	Desmodus rotundus	Costa Rica	Hernández-Mora G et al., mSphere 8:e00061-23.https://doi.org/10.1128/r
B. neosferati	bbatCR27	B. nosferati	Desmodus rotundus	Costa Rica	Hernández-Mora G et al., mSphere 8:e00061-23.https://doi.org/10.1128/r
B. neosferati	bbatCR26	B. nosferati	Desmodus rotundus	Costa Rica	Hernández-Mora G et al., mSphere 8:e00061-23.https://doi.org/10.1128/r
B. neosferati	bbatCR25	B. nosferati	Desmodus rotundus	Costa Rica	Hernández-Mora G et al., mSphere 8:e00061-23.https://doi.org/10.1128/r
B. neosferati	bbatCR22	B. nosferati	Desmodus rotundus	Costa Rica	Hernández-Mora G et al., mSphere 8:e00061-23.https://doi.org/10.1128/r
B. neosferati	bbatCR19	B. nosferati	Desmodus rotundus	Costa Rica	Hernández-Mora G et al., mSphere 8:e00061-23.https://doi.org/10.1128/r
B. neosferati	bbatCR18	B. nosferati	Desmodus rotundus	Costa Rica	Hernández-Mora G et al., mSphere 8:e00061-23.https://doi.org/10.1128/r
B. neosferati	bbatCR17	B. nosferati	Desmodus rotundus	Costa Rica	Hernández-Mora G et al., mSphere 8:e00061-23.https://doi.org/10.1128/r
B. neosferati	bbatCR16	B. nosferati	Desmodus rotundus	Costa Rica	Hernández-Mora G et al., mSphere 8:e00061-23.https://doi.org/10.1128/r
B. neosferati	bbatCR14	B. nosferati	Desmodus rotundus	Costa Rica	Hernández-Mora G et al., mSphere 8:e00061-23.https://doi.org/10.1128/r
B. neosferati	BCCN84.3	B. nosferati	Dog	Costa Rica	Guzmán-Verri C et al., Front Vet Sci. 2019;6:175.
Brucella core clade	REF Thomsen	B. suis bv 2	Ref. (Swine)	Denmark	Le Flèche et al., 2006
Brucella core clade	REF BOW 63-290	B. ovis	Ref. (Sheep)	Australia	Le Flèche et al., 2006
Brucella core clade	16M	B. melitensis bv 1	Ref. (Goat)		Jaÿ at al., 218, https://doi.org/10.3389/fvets.2018.00283
Brucella core clade	REF 16M	B. melitensis bv 1	Ref. (Goat)	USA	Le Flèche et al., 2006
